# Smart PZT-Embedded Sensors for Impedance Monitoring in Prestressed Concrete Anchorage

**DOI:** 10.3390/s21237918

**Published:** 2021-11-27

**Authors:** Quang-Quang Pham, Ngoc-Loi Dang, Jeong-Tae Kim

**Affiliations:** 1 Department of Ocean Engineering, Pukyong National University, Busan 48513, Korea; bkdn06x3a@gmail.com (Q.-Q.P.); dangngocloi@mtu.edu.vn (N.-L.D.); 2 Urban Infrastructure Faculty, Mien Tay Construction University, 20B Pho Co Dieu Street, Vinh Long 890000, Vietnam

**Keywords:** smart rebar, smart aggregate, impedance-based damage monitoring, sensitivity analysis, prestressed concrete anchorage

## Abstract

This study investigates the feasibility evaluation of smart PZT-embedded sensors for impedance-based damage monitoring in prestressed concrete (PSC) anchorages. Firstly, the concept of impedance-based damage monitoring for the concrete anchorage is concisely introduced. Secondly, a prototype design of PZT-embedded rebar and aggregate (so-called smart rebar–aggregate) is chosen to sensitively acquire impedance responses-induced local structural damage in anchorage members. Thirdly, an axially loaded concrete cylinder embedded with the smart rebar–aggregate is numerically and experimentally analyzed to investigate their performances of impedance monitoring. Additionally, empirical equations are formulated to represent the relationships between measured impedance signatures and applied compressive stresses. Lastly, an experimental test on a full-scale concrete anchorage embedded with smart rebar–aggregates at various locations is performed to evaluate the feasibility of the proposed method. For a sequence of loading cases, the variation in impedance responses is quantified to evaluate the accuracy of smart rebar–aggregate sensors. The empirical equations formulated based on the axially loaded concrete cylinder are implemented to predict compressive stresses at sensor locations in the PSC anchorage.

## 1. Introduction

In prestressed concrete (PSC) structures, anchorage zones resist particularly high compressive forces induced by pre-tensioned strands [1,2]. Due to the high local stress concentration initiated from steel-strands’ installation or overloading caused by natural disasters (e.g., earthquake events), incipient cracks regularly arise in the internal concrete body (often in the forms of micro-cracks) ahead of a bearing plate [3,4,5]. Since the bearing plates are commonly embedded in concrete blocks, incipient concrete damage is extremely difficult to detect. As inner cracks propagate to the concrete surface, the deterioration (e.g., strand corrosion or prestress-loss) could be severe under harsh environmental conditions. Therefore, the detection of damage occurrence in the PSC anchorage zone should be conducted to properly maintain the structural integrity of the PSC structures [6,7,8,9,10].

Currently, periodic visual inspection is commonly practiced for inspecting surface defects of concrete structures (e.g., spalling). Recently, vision-based monitoring has been applied to automatically identify crack or spalling in PSC structures via deep learning techniques [11,12]. However, the common inaccessibility (e.g., inner cracks in the anchorage zone) makes the visual-based inspection difficult and frequently inconclusive.

To overcome this limitation, various structural health monitoring (SHM) techniques have been developed by using sensing and analysis systems to enable real-time precautions. Vibration-based approaches have been successfully implemented to monitor structural defects in PSC structures by utilizing changes in vibration responses (e.g., shifts in natural frequencies) [13,14,15,16]. Attributed to the nature of lower-order modal parameters, however, global vibration-based approaches are obviously insensitive to local pristine structural damage. Some local inspection methods such as acoustic emission and X-rays have been examined for the health investigation of PSC members [17,18]. Strain-based methods via fiber optical sensor (FOS)-embedded smart strands are regarded as a practical way of detecting the variation of tensile forces in PSC members [19,20]. 

Among local SHM methods, impedance-based methods have been implemented for detecting various damage types, such as concrete damage [3,21,22], prestress-force quantification [23,24], the strength development of concrete [25,26]. For the technique, electromechanical (EM) impedance signals are gained from piezoelectric sensors (e.g., PZT, lead-zirconate-titanate) via coupling interactions of the PZT structure. Since impedance signatures characterize the structural features of the diagnosed zone, the change in structural properties leads to variation in impedance responses. That is, the local deterioration could be alarmed by computing variations in impedance signals measured at before and after a damaging event.

Research attempts have been made to monitor damage in a concrete structure using PZT patches affixed on its surface [27]. The impedance features measured from surface-bonded PZT sensors were strongly influenced by an environmental ambient, thereby demanding a complex algorithm to compensate for the environmental effects [28,29]. Additionally, many researchers have attempted to embed PZT sensors in concrete structures for monitoring curing-induced strength changes and hydration-induced stress variations [30,31,32]. In their works, brittle PZT sensors were commonly treated by protective layers to form small concrete blocks before embedding them into inspected structures. The feasibility of embeddable PZT sensors has been evaluated from a few applications.

As an alternative, a PZT sensor embedded in a concrete structure could be more suitable for monitoring impedance features sensitive to the occurrence of an incipient crack in a concrete body. There exist at least three reasons to develop PZT-embedded monitoring techniques for the PSC anchorage. Firstly, the PZT-embedded sensor can directly catch the variation of impedance signals induced by stress changes by knowing that the stress change yields the change in impedance responses [21]. Secondly, impedance features are less affected by environmental changes (e.g., temperature effects [28]) than surface-bonded sensors. Lastly, the PZT sensor is protected by coated layers (i.e., epoxy and concrete block), thus minimizing the sensor breakage and noise effect on signal acquisition [33,34]. Moreover, during the post-tensioning procedure, the impedance signatures of smart PZT sensors can be automatically measured and quantified via a statistical damage metric for each prestress-force level. Once concrete defects are detected, the actual load capacity can be re-determined by adjusting the prestress force in prestressing strands, thus ensuring structural integrity and minimizing maintenance costs. However, the implementation of the PZT-embedded transducers has not been rigorously evaluated for impedance-based damage in real-scale PSC anchorages.

As surveyed in the literature, the damage types in PSC structures can be classified as tendon corrosion, prestress loss, and concrete cracks. In this study, the target damage was selected to verify the proposed sensors. Therefore, the prestress-force variation during the installation of prestressing strands was selected to evaluate the practicability of a smart rebar and a smart aggregate. Firstly, the fundamentals of the impedance measurement via PZT-embedded sensors are concisely described for the anchorage zone. Secondly, two design samples of a PZT-embedded smart rebar and a PZT-embedded smart aggregate are selected to acquire the change of impedance responses sensitive to incipient defects in the PSC anchorage. Thirdly, numerical and experimental analyses are conducted on a concrete sample embedded with the smart rebar–aggregate to analyze its performance of impedance monitoring. For a sequence of loading cases, the change in impedance signatures is quantified using statistical RMSD (root mean square deviation) indices to analyze the sensitivity of the smart rebar–aggregate to the force changes. Lastly, an experimental test on a full-scale concrete anchorage embedded with smart rebar–aggregates at various locations is performed to evaluate the feasibility of the proposed method. For a sequence of loading cases, the variation in impedance responses is quantified to evaluate the accuracy of smart rebar–aggregate sensors. The empirical equations formulated based on the axially loaded concrete cylinder are implemented to predict compressive stresses at sensor locations in the PSC anchorage.

## 2. Impedance-Based Damage Monitoring for Prestressed Concrete Anchorage

### 2.1. Stress Responses of Anchorage Zone under Prestress Loading

**Figure 1:**
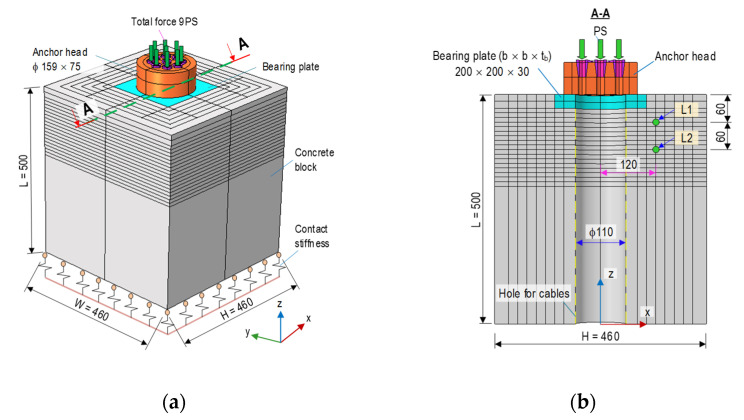
FE model of prestressed anchorage zone (dimension in mm): (**a**) 3D view of anchorage zone; (**b**) Cross-section A-A.

Figure 1 shows the finite element (FE) model of an anchorage zone with nine strands that was utilized to analyze the anchorage’s responses under prestressed forces. The FE model consists of an anchor head (φ = 159 for anchor-head diameter, H = 75 mm for anchor-head height), a bearing plate (b × b × t_b_ = 200 × 200 × 30 mm), and a concrete block (height × width × length = H × W × L = 460 × 460 × 500 mm). A duct (φ 110 mm) at the center of the concrete was used for passing steel-strands (see Figure 1b). The anchorage was designed to resist a minimum force of 1080 kN (120 kN per strand). The reinforcement for anchorage included a spiral (φ 10), orthogonal stirrups (φ 10), and longitudinal rebars (see details in Section 4.1). The compressive strength of concrete for the anchorage was *σ_c_* = 23.3 MPa (tested on a cylindrical sample with φ of 15 cm, and a height of 30 cm), and the tensile strength was selected as *σ_t_* = 2.33 MPa (0.1*σ_c_*). Moreover, the material properties of reinforcement were selected based on the recommendations of the company, VSL [35] (also listed in Table 2). The steel strands had a diameter of φ 15.2 mm, a breaking load of 260 kN, and a tensile strength of 1860 MPa. For the simplicity of the FE model, the reinforcement was converted into the equivalent modulus of concrete, and details of the FE model were presented by Dang et al. [36]. It is noted that the concept of converting reinforcement to equivalent modulus concrete could also be found in [37]. After converting, the material properties of equivalent concrete were $E_{eq}=29.89\;\mathrm{GPa}$ (modulus), $\sigma_{c}^{eq}=34.93\;\mathrm{MPa}$ (compressive strength), and $\sigma_{t}^{eq}=3.5\;\mathrm{MPa}$ (tensile strength).

**Figure 2:**
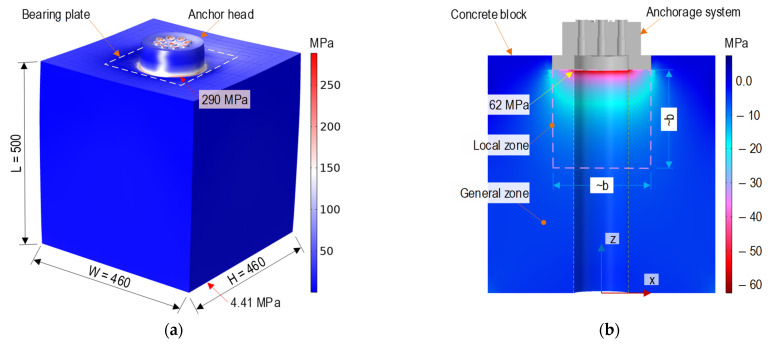
Stress distribution in prestressed anchorage zone subjected to applied force, PS: (**a**) von Mises stress; (**b**) Axial stress.

**Figure 3:**
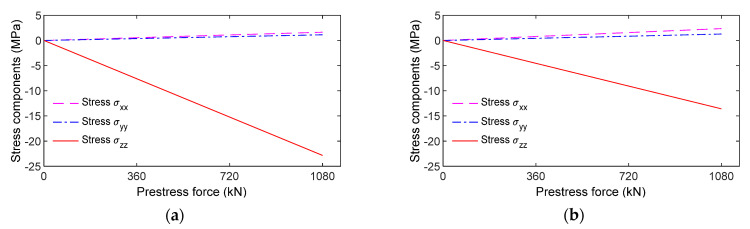
Stress responses versus applied force 0~9 PS in prestressed anchorage zone: (**a**) Point L1; (**b**) Point L2.

Figure 2a shows the von Mises stress in the anchor block induced by a total force of 9 PS (see Figure 1a). The maximum stress (about 290 MPa) occurred at the interfaces between the anchor head-bearing plates. The stress was nearly uniform stress (4.41 MPa) at the anchorage end. Figure 2b illustrates the axial stress on the concrete block with the highest compressive stress (about 62 MPa) at the contact surfaces between the concrete block-bearing plates. Figure 3a,b shows the relationships between the applied force (0~9 PS) and three stress components (*σ*_xx_, *σ*_yy_, and *σ*_zz_) examined at points L1 and L2 (its position can be seen in Figure 1b). The axial compressive stress (i.e., *σ*_zz_) at point L1 yielded a higher value (about 24 MPa) than that at point L2 (14.8 MPa). Furthermore, the lateral stresses (*σ*_xx_ or *σ*_yy_) at point L1 also experienced higher variation than those at point L2. It is known that von Mises stress is calculated from the root square of stress components (i.e., normal stresses and shear stresses), thus leading to this stress always having a positive value. The von Mises stress is commonly used to determine the yield states of ductile materials. For the anchorage zone, stress components were significantly different among them (see Figure 3). In this study, the von Mises stress was only used to visualize the stress distribution in the anchorage zone (see Figure 2a).

### 2.2. Theoretical Model of PZT-Embedded Smart Interface

#### 2.2.1. PZT-Embedded Smart Interface for Impedance Monitoring

**Figure 4:**
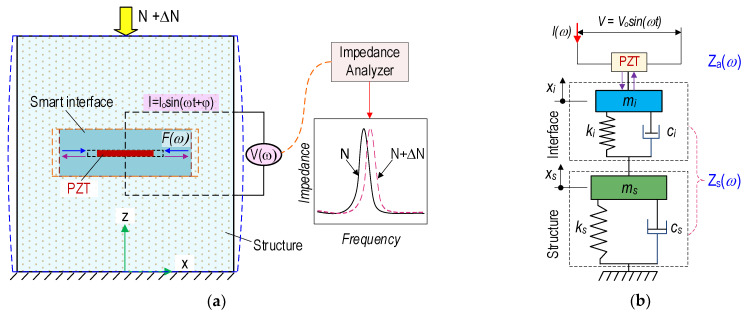
PZT-embedded smart interface for impedance monitoring in concrete structure: (**a**) Concrete structure embedded with a smart interface; (**b**) 2-DOF impedance model.

Figure 4a shows an interaction between the concrete structure and the PZT-embedded smart interface. The smart interface was fabricated by embedding a PZT patch into an interfacing media such as a rebar or a small concrete block. The smart interface was placed at desired locations in a monitored structure to acquire impedance responses using an impedance analyzer (see Figure 4a). 

Once the external load N alters (i.e., ΔN), it causes changes in the modal parameters (e.g., damping ratio) of the PZT interface and the host structure. As shown in Figure 4a, a mechanical strain represented by an external force *F*(*ω*) was produced due to the inverse piezoelectric effect under the applied voltage *V*(*ω*) to the PZT sensor. The force *F*(*ω*) was transferred into a local region of the inspected structure. Instantaneously, the structural response was transferred to the PZT patch to produce a voltage signal (the so-called direct piezoelectric effect). In practice, an output electric current, *I*(*ω*), was measured for the determination of EM impedance signals.

The EM impedance, *Z*(*ω*), is determined by the ratio of the input voltage and the output current [38]:

(1) $$Z(\omega )=\frac{V\left(\omega \right)}{I\left(\omega \right)}={\left\{j\omega A_{p}\left[{\hat{\varepsilon }}_{33}^{T}-\frac{1}{Z_{a}(\omega )/Z_{s}\left(\omega \right)+1}d_{31}^{2}{\hat{Y}}_{11}^{E}\right]\right\}}^{-1}$$

where *j*, ω, and *A_p_* are the imaginary unit, the excitation frequency, and the PZT-geometric parameters’ constant. *Z_a_* and *Z_s_* are the SM impedance of the PZT patch and one of the interface–host structures. The terms *d_31_*, ${\hat{\varepsilon }}_{33}^{T}$, and ${\hat{Y}}_{11}^{E}$ are the piezoelectric constant in one direction, the complex dielectric constant at zero stress, and the complex Young’s modulus of PZT patch at zero electric fields, respectively. Thus, the measured impedance response, *Z*(*ω*), represents the mechanical properties of the interface structure. Assuming that the structural mechanical (SM) impedance of the PZT-embedded interface is constant before and after the damaging events, changes in the structural properties can be determined by quantifying the variations of impedance responses (*Z*(*ω*)).

Figure 4b shows a two-degrees of freedom (2-DOF) impedance model, which represents the coupling SM interaction between the PZT-embedded interface–host structure [23]. One DOF stands for the motion of the interface, and the other stands for the motion of the host structure. The structural parameters include *m* (mass), *k* (damping coefficients), and *c* (spring stiffness), in which the symbols *i* and *s* represent the interface and the structure, respectively. 

Under a harmonic excitation *f_ext_*, the governing equation of motion at the PZT-driving point for the 2-DOF impedance model can be formulated as follows:

(2) $$\left[\begin{array}{cc} m_{i} & 0\\ 0 & m_{s} \end{array}\right]\left\{\begin{array}{c} {\ddot{x}}_{i}\\ {\ddot{x}}_{s} \end{array}\right\}+\left[\begin{array}{cc} c_{i} & -c_{i}\\ -c_{i} & c_{i}+c_{s} \end{array}\right]\left\{\begin{array}{c} {\dot{x}}_{i}\\ {\dot{x}}_{s} \end{array}\right\}+\left[\begin{array}{cc} k_{i} & -k_{i}\\ -k_{i} & k_{i}+k_{s} \end{array}\right]\left\{\begin{array}{c} x_{i}\\ x_{s} \end{array}\right\}=\left\{\begin{array}{c} f_{ext}\\ 0 \end{array}\right\}$$

where ${\ddot{x}}_{i},\;{\dot{x}}_{i},\;x_{i}$ and ${\ddot{x}}_{s},\;{\dot{x}}_{s},\;x_{s}$ are the accelerations, velocities, and displacements of the masses *m_i_* and *m_s_*, respectively. The coupling SM impedance, *Z_s_*, of the PZT interface and the host structure at the PZT driving point is determined as follows [23]:

(3) $$Z_{s}\left(\omega \right)=\frac{K_{11}\left(\omega \right)K_{22}\left(\omega \right)-K_{12}^{2}\left(\omega \right)}{j\omega K_{22}\left(\omega \right)}$$

where *K*_11_, *K*_12_, and *K*_22_ are the following dynamic stiffness coefficients *K*_11_ *= −ω*^2^*m_i_ + jωc_i_ + k_i_*, *K*_12_ *= −jωc_i_ − k_i_*, and *K*_22_ *= −ω^2^m_s_ + jω*(*c_i_ + c_s_*) *+* (*k_i_ + k_s_*). The stiffness coefficients depend on the structural parameters of the inspected structure and interface. Assuming there are no changes in the PZT patch electric and mechanical properties, any external effects (e.g., force variation) would cause changes in the measured impedance responses of the interface structure. Thus, any changes in the monitored structure can be quantified using the impedance features of the PZT-embedded interface.

#### 2.2.2. Impedance Monitoring Concept of Anchorage Zone Using PZT-Embedded Interface

**Figure 5:**
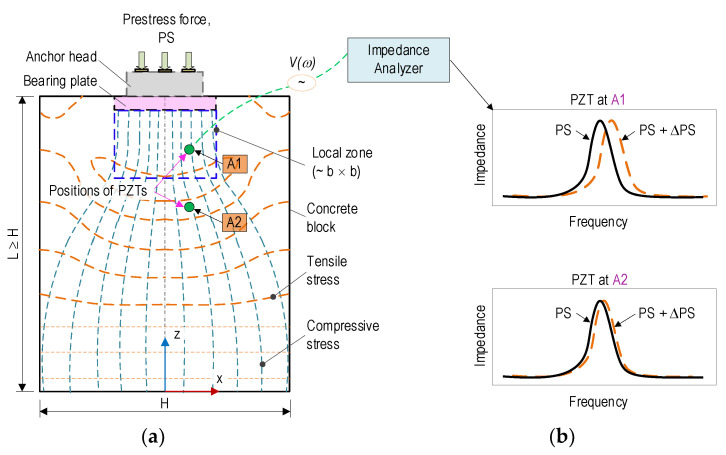
Concept of impedance-based damage monitoring in PSC anchorage via embedded PZT sensor: (**a**) PSC anchorage installed with PZT sensors; (**b**) Impedance responses obtained from PZTs.

Figure 5 shows the concrete anchorage zone (i.e., anchorage system and concrete block) of a PSC structure under prestress force, PS. The concrete block is potentially damaged due to the high-stress concentration [1,5]. Since the bearing plate of an anchorage system is commonly buried in the concrete block, incipient damage can occur inside a concrete block before spreading to the concrete surface [3,32].

As previously analyzed in Figure 1, Figure 2 and Figure 3, the anchorage zone’s stress fields caused by the prestress force can be examined in the local zone (ahead of the bearing plate) and the general zone [39,40]. The local zone, having a size of *b × b × b*, experiences particularly high compressive stress, in which *b* is the width of the bearing plate. Meanwhile, the general zone, having a size of L *×* H, undergoes a disturbance stress field induced by relatively high tensile and compressive stresses. At the end of the anchorage zone (where the anchorage length, L, is larger than the height, H), the stress fields are uniformly distributed for both tensile and compressive stresses. 

To monitor the impedance-responses-induced structural damage, the PZT-embedded smart interfaces can be placed at potential damage locations [41]. Figure 5a illustrates the locations of the two embedded PZT sensors in the concrete block with position A1 (in the local zone) and A2 (in the general zone). Figure 5b illustrates the impedance responses of the PZT sensors for the locations A1–A2, as the anchorage undergoes prestressing force variations. In general, the impedance features measured at location A1 would be more sensitive than those at location A2 due to the higher stress variation at location A1. The analysis demonstrated the potential application of PZT-embedded smart interfaces for damage monitoring in the anchorage zone.

Changes in the impedance signals between the intact state and a post-damage state are quantified using the following RMSD (root mean square deviation) index to characterize damage in the concrete anchorage [42]:

(4) $$RMSD\left(Z,Z^{*}\right)=\sqrt{\left(\sum_{i=1}^{N}{\left[Z^{*}(\omega_{i})-Z(\omega_{i})\right]}^{2}\right)/\sum_{i=1}^{N}{\left[Z(\omega_{i})\right]}^{2}}$$

where *Z*(*ω_i_*) and Z^*^(*ω_i_*) represent the real-part impedance signals obtained at the intact and damaged states at *i*th, a sweeping frequency in total measured frequency point *N*. In general, the RMSD magnitudes are only beyond zero when structural damage occurs. However, due to the uncertainties in experimental tests and computational error, the RMSD values could be greater than zero, even when no damage exists. To deal with the problem, a control limit (CL) is commonly used to assure the determination of damage existence [43], as follows:

(5) CL=μ + 3σ

where the mean value *μ* and the standard deviation *σ* of impedance signatures are calculated from an ensemble of the RMSD indices at the intact case. The CL with 3*σ* represents a damage identification with a confidence level of 99.7%.

## 3. Smart Rebar–Aggregate for Impedance-Based Damage Monitoring in PSC Structure

### 3.1. Design of Smart Rebar–Aggregate Sensors

**Figure 6:**
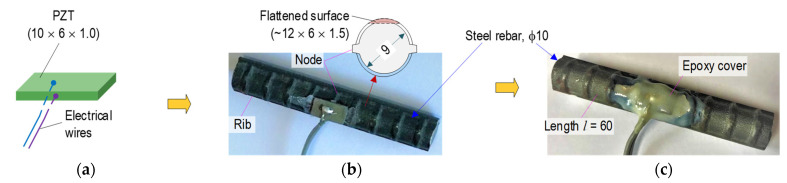
Prototype of PZT-embedded rebar for impedance monitoring (unit in mm): (**a**) PZT patch; (**b**) PZT mounted on steel rebar, (**c**) Smart rebar.

**Figure 7:**
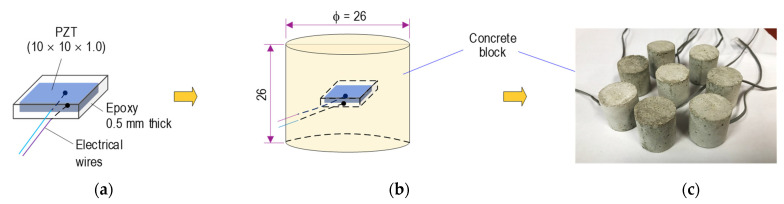
Prototype of PZT-embedded aggregate for impedance monitoring (unit in mm): (**a**) Covered PZT patch; (**b**) PZT embedded in concrete block, (**c**) Smart aggregate.

Figure 6 and Figure 7 show the two types of PZT-embedded interfaces (so-called smart rebar and smart aggregate sensors) designed for acquiring changes in impedance-responses-induced structural damage in the concrete structure. For the application to concrete structures, the PZT sensors should be formed as suitable interfaces considering the durability of the PZT patch and the sensitivity of signal acquisition. Considering the fragility of the PZT patches, the PZT patches should be properly protected before embedding their anchorage structure for an impedance measurement. In this study, the following two forms of PZT sensors were selected: a PZT-embedded aggregate [3,44] and a PZT-embedded rebar [45].

As shown in Figure 6, a prototype of the PZT-embedded rebar (SR) was selected for acquiring impedance signatures in concrete structures. A 5A-PZT patch (10 × 6 × 1 mm) was soldered with an electrical wire for gaining impedance responses (see Figure 6a). The PZT patch was attached to the steel rebar (see Figure 6b) using high-strength quick glue (Loctite 401). Then, the PZT sensor was covered by an epoxy layer with a machined rebar surface (12 × 6 × 1.5 mm) for insulating electricity and waterproofing. The rebar φ 10 mm (Korean standard) had nodes (thickness of 1 mm) and two ribs used in the test. Figure 6c shows a sample of the smart rebar 12 h after coating the epoxy layer.

As shown in Figure 7, a prototype of the PZT-embedded smart aggregate (SA) was designed for impedance monitoring. A PZT 5A patch, with a size of 10 × 10 × 1 mm, was welded with an electrical wire, and it was then coated by a 0.5-mm-thick epoxy (see Figure 7a). The protected PZT sensor was positioned in a small concrete block, which had the size of φ 26 mm and a height of 26 mm (see Figure 7b). Figure 7c shows the smart aggregate samples after casting for 28 days. As listed in Table 1, a mixture of concrete including sand, cement, and water (excluding coarse aggregate D_max_ 25) was utilized to make the smart aggregate sensors. 

**Figure 8:**
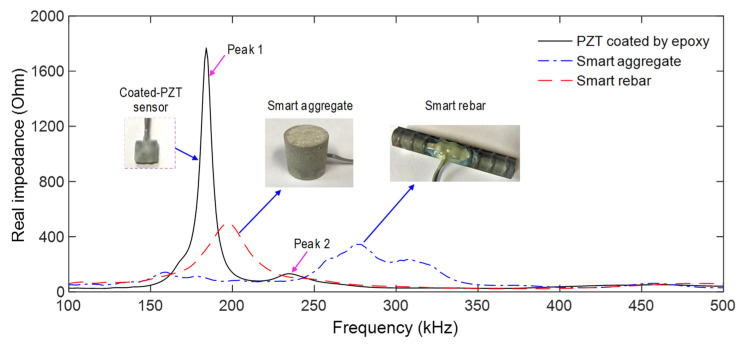
Impedance signatures of coated PZT sensor, smart rebar, and smart-aggregate.

Figure 8 shows the measured impedance signatures for a coated PZT sensor, a PZT-embedded rebar (after 12 h of epoxy coating), and a PZT-embedded aggregate (after 28 days of curing). The effects of the fabrication media and process were examined on the sensitivities of the impedance signatures. The first resonant impedance frequency of the coated-PZT was about 190 kHz. After being formed into the PZT-embedded aggregate, the first impedance frequency was shifted rightward to about 200 kHz, and the impedance magnitude was significantly decreased. Moreover, after being formed into the PZT-embedded rebar, the first impedance frequency was also shifted rightward (about 285 kHz) and its magnitude was decreased due to the damping effect of the epoxy layer [46].

### 3.2. Numerical Analysis of Smart Rebar–Aggregate for Impedance Monitoring

#### 3.2.1. Finite Element Model of Smart Rebar–Aggregate

**Figure 9:**
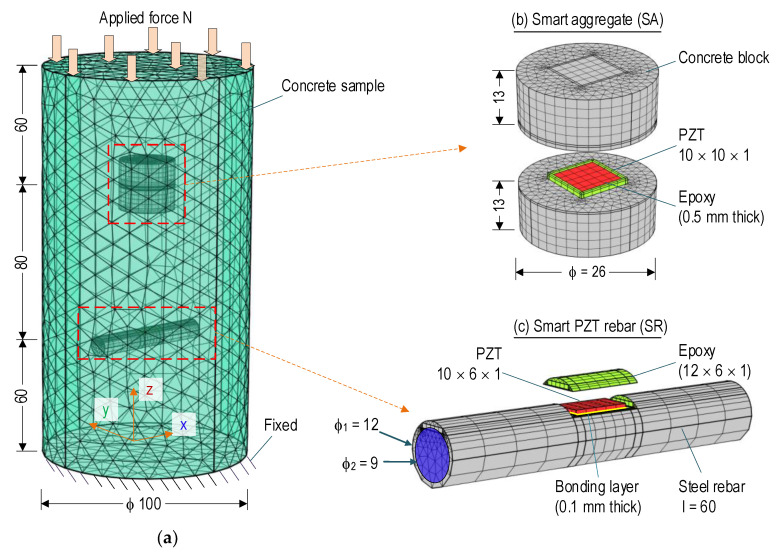
FE model of smart rebar–aggregate under compressive force (unit in mm): (**a**) Rebar–aggregate in concrete sample; (**b**,**c**) Detailed meshing at rebar–aggregate.

The sensitivity of signal acquisition was numerically investigated for the smart sensing interfaces (i.e., PZT-embedded rebar and PZT-embedded aggregate), as seen in Figure 9. An FE model of a cylindrical concrete embedded with the SR and the SA was constructed to simulate impedance signatures under compressive loading cases. The concrete sample had φ of 100 mm and height of 200 mm, and the concrete properties are defined in Table 2.

The FE model was meshed with 32,319 elements (26,030 elements for the concrete component (see Figure 9a), 3679 elements for the SA (see Figure 9b), and 2610 elements for the SR (see Figure 9c). As shown by zoomed-in view in Figure 9b, the meshed SA consisted of 3532, 122, and 25 elements for the concrete, the epoxy layer, and the PZT patch, respectively. As shown by the zooming in view in Figure 9c, the meshed SR comprised of 2281, 279, and 25 elements for the steel rebar, the epoxy layer, and elements for the PZT patch, respectively. To ease the continuity among the meshed parts of the FEM, the quadratic hexahedron elements were utilized for the PZT patch and epoxy of the SA and the PZT patch of the SR, and the remaining parts used quadratic tetrahedron elements. For the simulation of impedance responses, the damping loss factor (for concrete, steel, epoxy, and the bonding layer) and the dielectric loss factor (for piezoelectric materials) were selected as η = 0.0125 and δ = 0.015 [47]. A fixed boundary condition was assigned to one face of the concrete (see Figure 9a).

The SA (φ of 26 mm, and height of 26 mm) and the SR (length *l* = 60 mm, φ_2_ = 9 mm) were placed at the center of the concrete sample, and their distance to each end of the concrete surface was 60 mm. To simplify the FEM of the SR, the nodes and ribs of the steel bar (see Figure 6b) were simulated as solid elements with an outer diameter φ_1_ = 12 mm, as seen in Figure 9c. A bonding layer between the PZT patch-rebar had a thickness of 0.1 mm. The geometric constants and material characteristics of the concrete block of the SA (see Figure 9b), PZT 5A, and the epoxy layers are described in Section 3.1 and also listed in Table 2. The material properties of the cylindrical concrete (see Figure 9a), which was the host structure, were selected as the same as those of the SA (see Table 2). 

**Figure 10:**
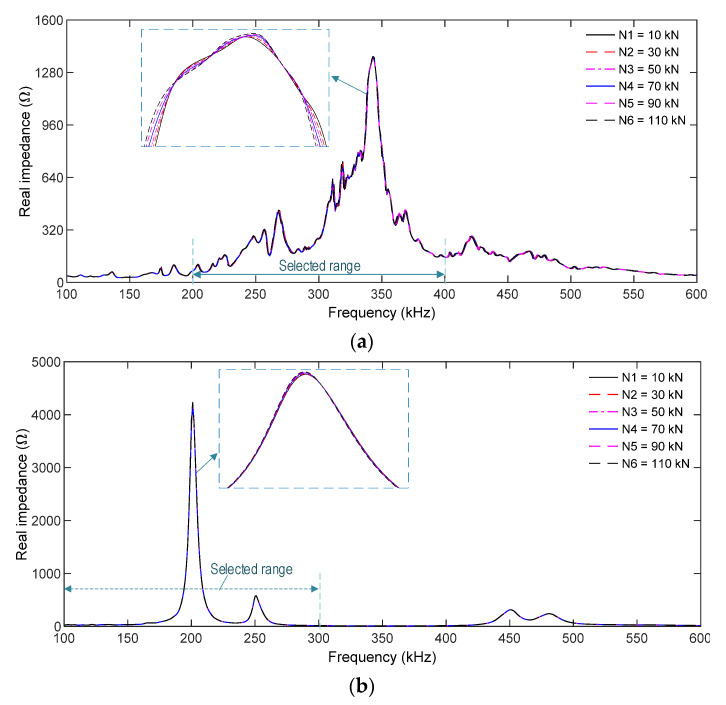
Numerical impedance responses of PZT sensors in the concrete sample under compression: (**a**) PZT-embedded smart rebar (SR); (**b**) PZT-embedded smart aggregate (SA).

Six loading cases, namely N1–N6, were simulated to gain impedance responses. The compressive force was gradually increased in a row from 10 (N1) to 110 kN (N6) with an interval of 20 kN (see Figure 10), corresponding to the axial stress from 1.3 (N1) to 14.0 MPa (N6). The impedance signatures of the PZT sensors were simulated in the range 100–600 kHz with an interval of 1.0 kHz points by applying 1V harmonic excitation to the top surface of the PZT patch, and the bottom surface was assigned with the ground electrode.

#### 3.2.2. Numerical Impedance Features of Smart Rebar–Aggregate under Compression

Figure 10a,b, respectively, show the impedance responses of the SR and the SA in the simulated range of 100–600 kHz for the load cases N1–N6. Several resonant impedance signals were found in the simulated frequency range for the two PZT sensors. The main resonant frequency peak was about 340 kHz for the PZT-embedded rebar (see Figure 10a) and 200 kHz for the PZT-embedded aggregate (see Figure 10b). Under the loading cases, the variations in the impedance responses of the PZT rebar were slightly higher than those of the PZT-embedded aggregate. 

**Figure 11:**
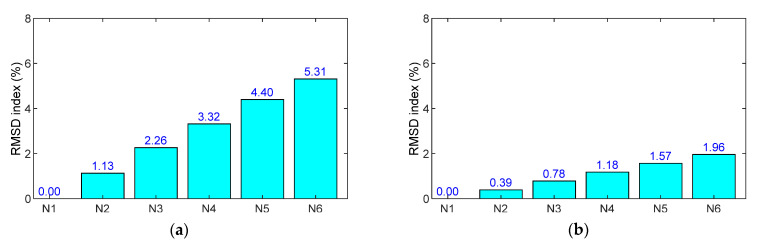
RMSD indices of numerical impedance signals of PZT sensors in the concrete sample under compression: (**a**) PZT-embedded smart rebar (SR); (**b**) PZT-embedded smart aggregate (SA).

As resonant impedance signals comprise meaningful structural parameters at the PZT-driven point [48,49], the frequency ranges 200–400 kHz for the SR and 100–300 kHz for the SA were selected to quantify impedance-responses-induced force changes. As shown in Figure 11a,b, the RMSD magnitudes of PZT sensors linearly increased with respect to the applied forces. The RMSD of the PZT-embedded rebar shows a higher sensitivity than that of the PZT-embedded smart aggregate. In the case of N6 (110 kN), the RMSD index was 5.31% for the SR and 1.96% for the SA, indicating that the smart rebar has a higher sensitivity to impedance monitoring in the concrete anchorage.

### 3.3. Experimental Analysis of Smart Rebar–Aggregate for Impedance Monitoring

#### 3.3.1. Test Setup of Smart Rebar–Aggregate

**Figure 12:**
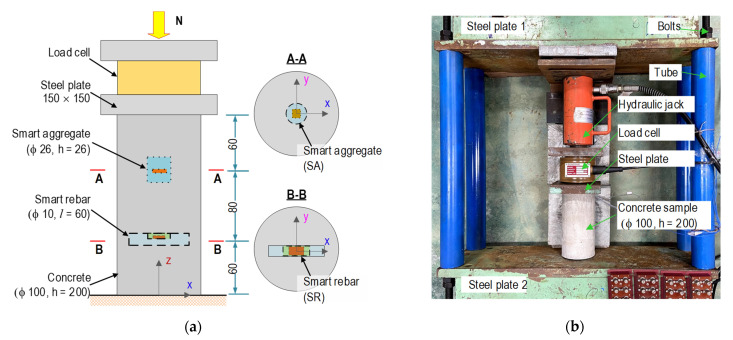
Test setup on a concrete sample for impedance measurement (dimension in mm): (**a**) Orientation of PZT sensors; (**b**) Concrete sample on a test structure.

The smart rebar–aggregate was experimentally evaluated on a concrete sample embedded with the smart interfaces, as schematized in Figure 12. The concrete sample had φ of 100 mm and a height of 200 mm (the same geometry as in the FE model). The mixture for the concrete sample was selected in Table 1 with *σ_c_* = 23.3 MPa (compressive strength), as listed in Table 2.

Figure 12 shows the concrete sample positioned with two prototypes of the SR and the SA at the center of the test sample. The steel rebar was 60 mm in length, in which the PZT sensor was surface bonded on the middle section. The SR and the SA were positioned 60 mm from two ends of the concrete block. Moreover, the orientation of the PZT sensors in the concrete sample is shown in cross-sections A-A and B-B (see Figure 12a). The geometric parameters and fabrication process of the SR and the SA were detailed in the previous section. Moreover, the material characteristics of the concrete sample, steel rebar, epoxy adhesive, and PZT 5A are listed in Table 2.

Figure 12b shows the test setup of the concrete sample on a steel frame, which was used to control the compressive forces introduced by a hydraulic jack. Six test cases (N1–N6) were simulated for impedance measurements. The compressive force was gradually increased from N1 (10 kN) to N6 (110 kN) with a 20 kN increment, corresponding to 1.3–14.0 MPa (N1–N6). For the impedance measurements, HIOKI 3532 (impedance machine) was utilized to produce 1 V of excitation and acquire signatures using the 100–600 kHz (501 measured points) frequency range. For tests N1–N6, the impedance signals were recorded for an ensemble with four measurements to calculate the control limit (see Equation (5)) and check the stability of the measured data. Furthermore, the effects of temperature variation on the impedance features were minimized by maintaining room temperatures at around 21 °C (using conditioners) during the measuring process.

#### 3.3.2. Experimental Impedance Features of Smart Rebar–Aggregate under Compression

**Figure 13:**
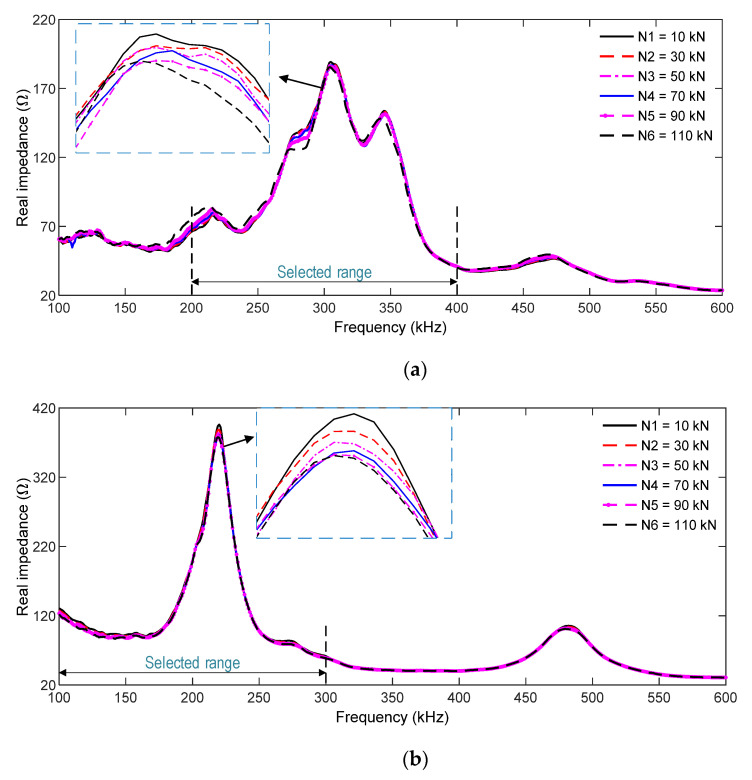
Experimental impedance responses of PZT sensors in the concrete sample under compression: (**a**) PZT-embedded smart rebar (SR); (**b**) PZT-embedded smart aggregate (SA).

Figure 13 shows the impedance responses of the SR and the SA (100–600 kHz) for six loading cases. The impedance signatures were slightly varied with respect to an increasing compressive force, but their changes were insignificant. There were resonant impedance signals with resonant peaks as follows: about 300 kHz for the SR (see Figure 13a) and 210 kHz for the SA (Figure 13b). The frequency ranges of 200–400 kHz (for the SR) and 100–300 kHz (for the SA) were selected for impedance-based damage monitoring.

**Figure 14:**
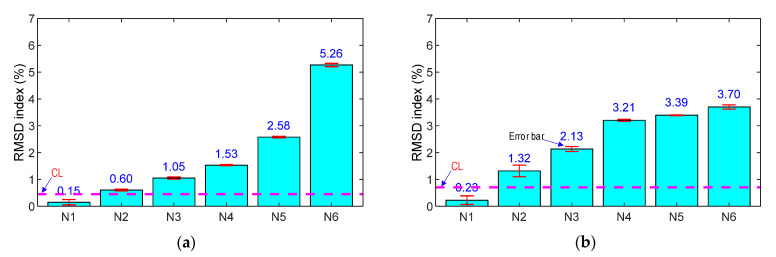
RMSD indices of experimental impedance signals of PZT sensors in the concrete sample under compression: (**a**) PZT-embedded smart rebar; (**b**) PZT-embedded smart aggregate.

The variations in impedance signals under the loading cases were quantified using the RMSD damage metric, as depicted in Figure 14a,b. In addition, the CL (control limit) was computed using four ensembles of impedance signals (200–400 kHz for the SR and 100–300 kHz for the SA) in the intact case. The standard deviation of the measured data was also examined for the loading cases (see error bars in the figures). The RMSD values were negligible under the intact case (<0.5%), but these values were beyond the CL control threshold for the loading cases. Moreover, the dispersion of the data was relatively low, as seen in the error bars in the figures. This indicated that the force or stress variations (about 20% corresponding to two successive cases) in the concrete sample were successfully detected by both the smart rebar and the smart aggregate.

**Figure 15:**
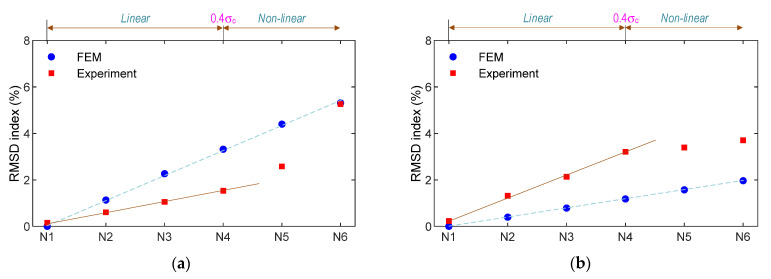
Comparison of numerical and experimental RMSD indices of impedance signals: (**a**) PZT-embedded smart rebar; (**b**) PZT-embedded smart aggregate.

As shown in Figure 15, the relationship between the applied force versus the RMSD indices was analyzed for the numerical and experimental impedance signals of the smart rebar–aggregate. Under the force variation, the RMSD magnitude of the PZT rebar (see Figure 15a) was relatively higher than that of the PZT-embedded aggregate (see Figure 15b). Moreover, under the same axial force, the RMSD magnitude of the PZT rebar was larger than that of the smart aggregate for the numerical impedance feature. Meanwhile, for the experimental impedance feature, the RMSD magnitude of the PZT rebar was only greater than that of the SA for the force larger than 90 kN (N5). It is noted that the stress variation of the steel rebar was higher than that of the concrete block. Consequently, the higher stress variation in the PZT rebar caused the higher variation in impedance features [43,48]. For both the smart rebar and the smart aggregate, there were differences in the RMSD magnitudes of the numerical and experimental signals. This could have been caused by prestressing effects on the coated PZT sensor during concrete strength development [3] and non-linear behaviors of the materials (which was not considered in the FE model). 

For both the SR and the SA, the relationships between the compressive loads (N1–N6) and the RMSD damage metrics were linear once the force was lower than 70 kN (about 0.4σ_c_). This observation is consistent with the behavior of the normal concrete [50,51]. When the stress in the concrete sample was larger than 0.4σ_c_, the RMSD magnitudes of the smart rebar rapidly increased, that is, the RMSD index at loading N6 (i.e., 5.26%) was larger than that of loading N5 (i.e., 2.58%). Meanwhile, the RMSD value of the smart aggregate slightly increased, as shown in Figure 15b.

**Figure 16:**
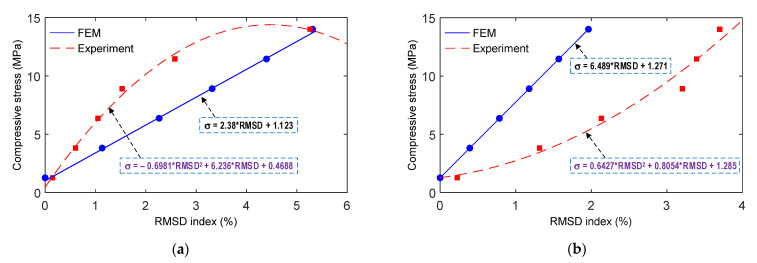
Empirical formulations of measured impedance signatures with respect to applied compressive stresses: (**a**) PZT-embedded smart rebar; (**b**) PZT-embedded smart aggregate.

Empirical equations of the measured impedance signals with respect to the applied compressive stresses were quantified for the smart rebar and the smart aggregate. Figure 16a,b show empirical equations of the smart rebar and the smart aggregate, respectively, to predict compressive stresses via the RMSD indices of the impedance signals. For both the smart rebar and the smart aggregate, the FEM results (Equation (6) for the SR and Equation (8) for the SA) exhibit simple linear regressions, and the experimental results (Equation (7) for the SR and Equation (9) for the SA) exhibit second order polynomial regressions on RMSDs versus stresses, as indicated in Figure 16. These derived empirical equations (i.e., Equations (6)–(9)) would be used to predict applied compressive stresses in the target anchorage structure under loading cases.

(6) σ=2.38RMSD+1.123

(7) $$\sigma =-0.698RMSD^{2}+6.236RMSD+0.469$$

(8) σ=6.489RMSD+1.271

(9) $$\sigma =-0.643RMSD^{2}+0.805RMSD+1.285$$

## 4. Evaluation of Smart Rebar–Aggregate for Real-Scale PSC Anchorage

### 4.1. Description of PSC Anchorage with Smart Rebar–Aggregate

#### 4.1.1. Design of PSC Anchorage

**Figure 17:**
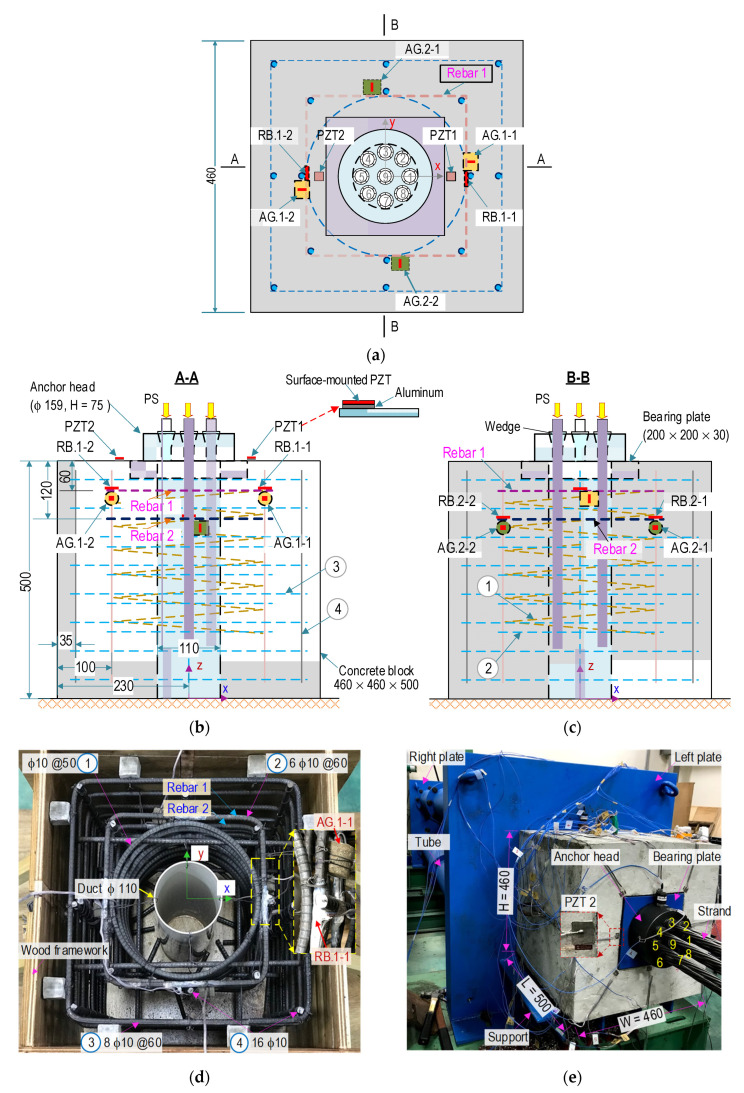
Diagram of PSC anchorage zone installed with smart rebar–aggregates (dimension in mm): (**a**) Top-view of anchorage configuration; (**b**) Cross-sectional A-A; (**c**) Cross-sectional B-B; (**d**) Reinforcement and sensor installation; (**e**) Experimental setup of PSC anchorage.

To assess the practicability of the smart rebar–aggregate sensors for impedance monitoring, a multi-strands concrete anchorage embedded with SRs and SAs was conducted, as systematized in Figure 17. The anchorage zone consists of a nine-strand anchorage head (φ of 159 mm and a height of 75 mm) with wedges, a steel bearing plate (20 × 20 × 3 cm), and a reinforced concrete block (46 × 46 × 50 cm). As shown in Figure 17a–c, the geometric parameters of the concrete block were selected based on VSL’s recommendation [35]. The concrete block was designed with reinforcements to resist a minimum compressive force of 1080 kN (120 kN per prestressing strand). The reinforcement shown in those figures was selected as follows: (1) spiral φ 10, spacing @ = 50 mm, and *l* = 4890 mm; (2) orthogonal stirrups 6 φ 10, @ = 60 mm, and *l* = 1280 mm; (3) orthogonal stirrups 8 φ 10, @ = 60 mm, and *l* = 1760 mm; and (4) longitudinal rebars 16 φ 10 and *l* = 820 mm (see Figure 17b–d). A PVC (polyvinyl chloride) cylinder (φ 110 mm) was placed at the central concrete block for passing steel strands.

The concrete mix for the concrete block (*σ_c_* = 23.3 MPa at 28-day) are listed in Table 1. Moreover, the material characteristics of reinforcement and concrete were previously presented in Table 2. For the anchorage components, (i.e., anchor head, wedges, and bearing plate), their material characteristics were defined as follows: *ρ* = 7850 kg/m^3^ (mass density), *ν* = 0.33 (Poisson’s ratio), and *E* = 200 GPa (elastic modulus). The seven-wire strands with φ of 15.2 mm (nominal diameter) and a length of 6.0 m (Grade 270 low-relaxation steel, 140 mm^2^ in cross-section area) had the following material properties: *E* = 195 GPa (Young’s modulus) and *F_pk_* = 260.4 kN (breaking load).

Figure 17d shows the arrangement of the reinforcement and the sensor during the construction of the concrete anchorage. Figure 17e shows the installation of the anchorage zone (i.e., anchorage system, concrete block, and prestressing strand) on a steel frame. The steel frame, including four thick steel tubes used to connect two thick steel plates, can resist a 3000 kN compressive force induced by prestressing strands. On the dead-end (i.e., left steel plate), strands passing two holes on the anchor block (φ 110 mm) and the steel plate (φ 145 mm) were gripped into a multi-strand anchor head. On the live-end (i.e., right steel plate), the strands were distributed into holes (150 mm in the distance) and connected to hydraulic jacks. The jacking systems were used to control tensions in the prestressing strands. Real tensions in the prestressing strands were measured via load cells installed on the right-ends strands.

#### 4.1.2. Deployment of PZT-Embedded Smart Sensor

To acquire the impedance signals of the tested structure under prestressing force variations, the following three types of PZT sensors were deployed: PZT-embedded smart rebars, PZT-embedded smart aggregates, and surface-mounted PZT sensors. As shown in Figure 17a–c, two smart rebars, namely RB.1-1 and RB.1-2, were installed on Rebar one (see Figure 17a,b), and two others, namely RB.2-1, and RB.2-2 were installed on Rebar two (see Figure 17c). The distances from Rebar one and two to the top anchorage surface were about 60 and 120 mm, respectively. The geometric constants and material characteristics of those sensors were detailed in the previous sections. 

As shown in the figures, two smart aggregates, namely AG.1-1 and AG.1-2, were installed on Rebar one (see Figure 17a,b), and two smart aggregates, namely AG.2-1 and AG.2-2), were attached on Rebar two (see Figure 17c). Additionally, two surface-mounted PZT sensors (i.e., PZT one and PZT two) were installed on the anchorage surface (see Figure 17a,b). To enhance the impedance responses of PZTs 1–2, PZT 5A (10 × 10 × 0.5 mm) patches were mounted via an aluminum plate (10 × 10 × 0.5 mm) [52]. Loctite 401 (instant adhesive) was used to glue the PZT sensors on the aluminum plates and the aluminum plates to the concrete surface.

### 4.2. Description of PSC Anchorage with Smart Rebar–Aggregate

#### 4.2.1. Experiment Scenario

Four cases of prestress force, namely PS1–PS4, were conducted for acquiring impedance signals. For the first test case (PS1), each of the nine strands were separately pre-tensioned with a small force (about one kN) to set up the prestressing strands and anchorage zone. As the intact baseline for the experiment, all the load cells were initialized by setting zero force (0 kN). In the second test case (PS2), each of the nine strands were tensioned up to about 40 kN. The sequence of tensioning strands was conducted as follows: Strand nine (the center strand), Strands one and five (two outer strands), Strands three and seven, Strands two and six, and Strands four and eight (see Figure 17e). In the third test case (PS3), each of the strands were tensioned up to about 80 kN. In the last test case (PS4), the strands were pre-tensioned to about 120 kN.

The HIOKI 3532 impedance analyzer was utilized to measure the impedance responses of the PZT sensors using an exciting range of 100–600 kHz (an interval of 501 points). Four ensembles of impedance signals were recorded for each test case (PS1–PS4) to calculate. During the impedance measurement, the laboratory temperatures were kept at about 21.5 °C (air conditioners) to minimize the temperature effects on the impedance features. 

#### 4.2.2. Impedance Responses of PZT-embedded Sensors for Intact Case (PS1)

**Figure 18:**
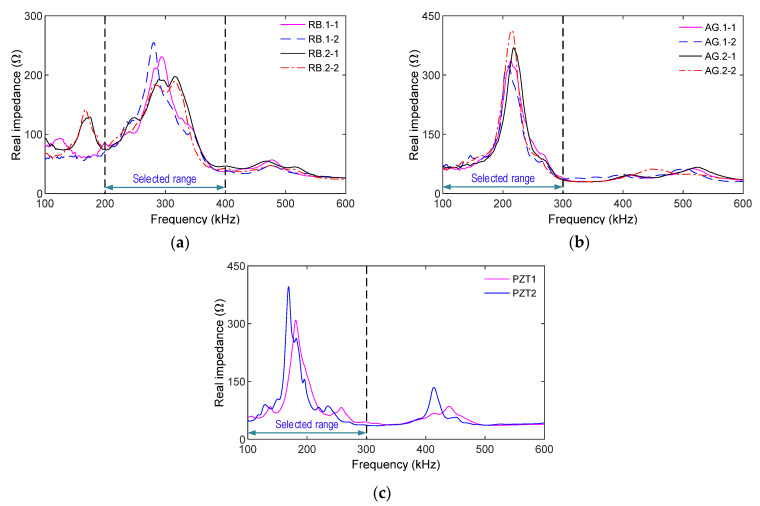
Impedance responses of PZT-embedded sensors for intact case (PS1): (**a**) PZT-embedded smart rebar; (**b**) PZT-embedded smart aggregate; (**c**) Surface-bonded PZT sensor.

Figure 18 shows the impedance signatures (100~600 kHz frequency range) measured at the intact state (PS1) via the embedded and surface-mounted PZT sensors. For all the smart rebars (i.e., RB.1-1~RB.2.2), high resonant impedance responses were found in the range of 200~400 kHz with the resonant frequency around 300 kHz (see Figure 18a). For the four smart aggregates (i.e., AG.1-1~AG.2.2), resonant impedance signals were found in 100~300 kHz with the resonant frequency around 210 kHz (see Figure 18b). For the surface-bonded sensors (i.e., PZTs 1-2), resonant impedance signals were found in 100–300 kHz with the resonant frequency around 190 kHz (see Figure 18c). Those impedance peaks represent the information on the structural behaviors of the inspected structure; therefore, the impedance ranges were selected to monitor the anchorage model.

#### 4.2.3. Impedance Signatures of PSC Anchorage under Prestressing Force Variations

**Figure 19:**
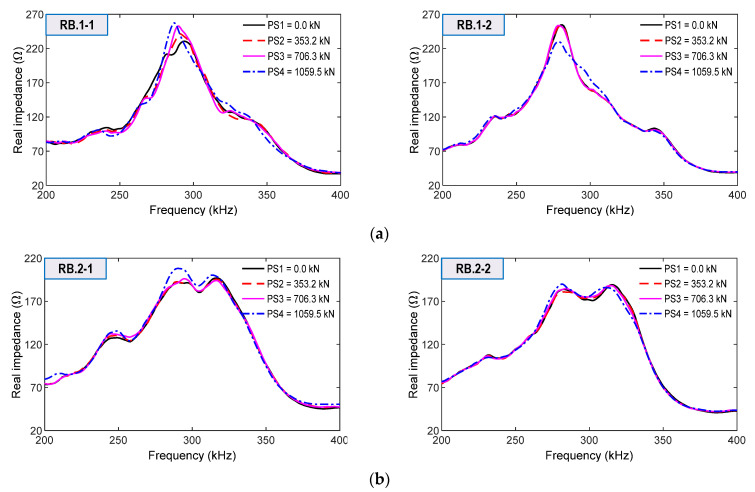
Impedance signatures of smart rebars for loading cases PS1–PS4: (**a**) Smart rebars RB.1-1 and RB.1-2 on Rebar 1; (**b**) Smart rebars RB.2-1 and RB.2-2 on Rebar 2.

From the smart rebars, impedance signals were measured in the range 200–400 kHz for the loadings PS1~PS4, as shown in Figure 19a,b. The impedance responses of RB.1-1 and RB.1-2 on Rebar one (close to the bearing plate) were relatively more sensitive to the force variations than those of RB.2-1 and RB.2-2 on Rebar two (located at 60 mm below Rebar one). Among the four sensors, RB.1-1’s impedance signals were the most sensitive, as indicated in Figure 19a. 

**Figure 20:**
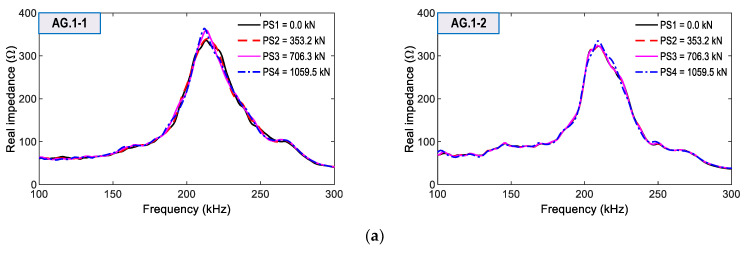

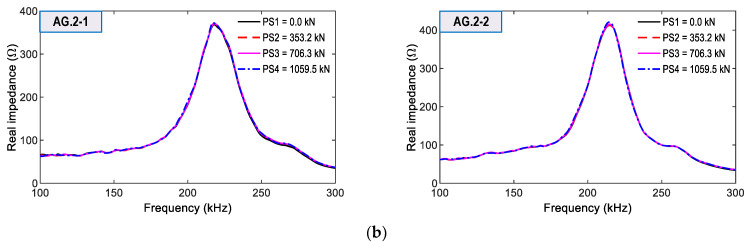
Impedance signatures of smart aggregates for loading cases PS1~PS4: (**a**) Smart aggregates AG.1-1 and AG.1-2 on Rebar 1; (**b**) Smart aggregates AG.2-1 and AG.2-2 on Rebar 2.

From the four smart aggregates, the impedance signals were measured in the range of 100–300 kHz for the same loading cases. As shown in Figure 20a,b, the impedance responses of AG.1-1 and AG.1-2 (attached on Rebar one) were comparatively more sensitive to prestressing force variation than those of AG.2-1 and AG.2-2 (attached on Rebar two). Moreover, the impedance signals of AG.1-1 showed the highest sensitivity among the four smart aggregates. This observation was consistent with the impedance responses obtained from the smart rebar RB.1-1. In addition, the impedance signals of AG.2-1 and AG.2-2 on Rebar two had ignorable variations under the prestressing cases.

**Figure 21:**
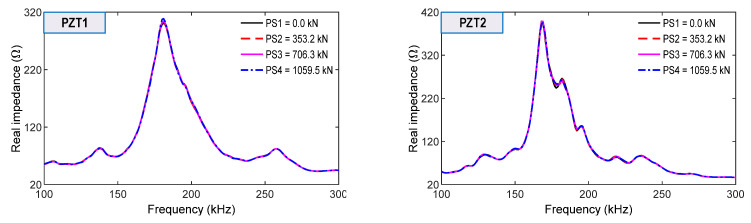
Impedance signatures of surface-mounted PZT sensors for loading cases PS1~PS4.

From the surface-bonded PZT1 and PZT2, impedance signals were measured in the range of 100–300 kHz for the loading cases (see Figure 21). The changes in the impedance signals measured on the concrete surface were insignificantly small compared to those by the smart sensors inside the anchorage.

### 4.3. Evaluation of Smart Rebar–Aggregate Sensing in PSC Anchorage

#### 4.3.1. Sensitivity of Smart Rebar–Aggregate under Prestress-Force Variation

**Figure 22:**
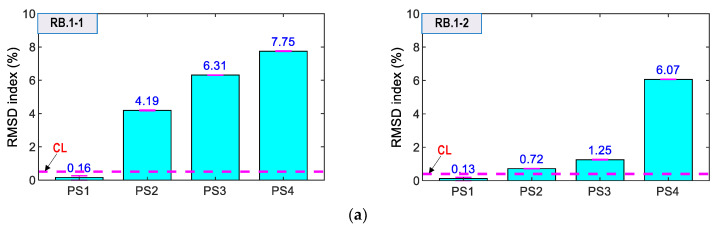

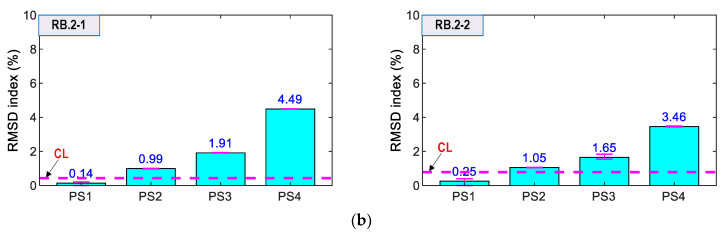
RMSD indices of impedance signals via smart rebars under loading cases PS1~PS4: (**a**) Smart rebars RB.1-1 and RB.1-2; (**b**) Smart rebars RB.2-1 and RB.2-2.

**Figure 23:**
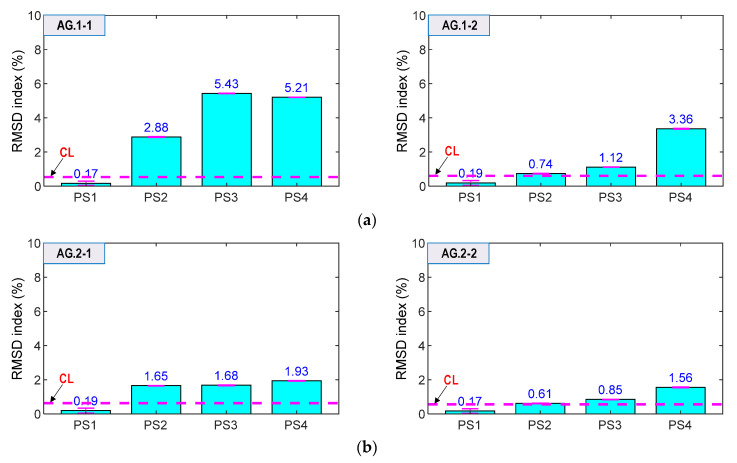
RMSD indices of impedance signals via smart aggregates under loading cases PS1~PS4: (**a**) Smart aggregates AG.1-1 and AG.1-2; (**b**) Smart aggregates AG.2-1 and AG.2-2.

**Figure 24:**
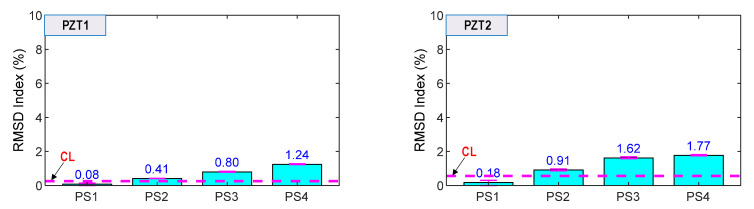
RMSD indices of impedance signals via surface-mounted under loading cases PS1~PS4.

The statistical damage metric RMSD was utilized to quantify the variations in the impedance responses for loading cases. The ensembles of signals at the first test cases (intact case) were utilized to calculate the CL for each sensor. For each of the prestressing cases, PS2~PS4, four ensembles of measured data were used to evaluate the deviation of the impedance data. For the calculation of the RMSD indices, the resonant frequency ranges, selected as 200–400 kHz for the smart rebars, 100–300 kHz for the smart aggregate, and 100–300 kHz for the surface-bonded sensors, were utilized, as shown in Figure 22, Figure 23 and Figure 24.

As shown in Figure 22, the RMSD magnitudes of RB.1-1, RB.1-2, RB.2-1, and RB.2-2 were relatively small (<1.0%) for the intact state (i.e., PS1), and they were lower than the control thresholds. For loading cases PS2–PS4, the RMSD magnitudes were increased and over the thresholds, thus suggesting that the variation of prestress forces was successfully alarmed. Moreover, the standard deviations of the measured impedance signals were very small (see Figure 22a,b). Specifically, the RMSD values of the sensors on Rebar one (i.e., RB.1-1 or RB.1-2) were higher than those of the sensors on Rebar two (i.e., RB.2-1 or RB.2-2). The result confirmed that Rebar two (further to the bearing plate) experienced less stress changes than Rebar one [1,53]. We found that smart rebars should be placed in Rebar one to sensitively catch the impedance-responses-induced force variation, due to higher stress changes causing more variations in the impedance features [21,48].

As shown in Figure 23, the RMSD values of AG.1-1, AG.1-2, AG.2-1 and AG.2-2 were ignorable (<0.6%) for the intact state (PS1). Meanwhile, the magnitudes were increased and beyond the thresholds for the cases PS2–PS4. Additionally, the error bars (see in figures) were relatively small. The result suggested that the smart aggregates successfully alarmed the prestressing force variation. Moreover, the RMSD magnitudes of AG.1-1 and AG.1-2 on Rebar one (see Figure 23a) were relatively higher than those of AG.2-1 and AG.2-2 on Rebar two (see Figure 23b). This observation was consistent with the results of the smart rebars.

As shown in Figure 24, the RMSD indices of the surface-bonded PZT sensors (i.e., PZT1 and PZT2) were ignorable (<0.5%) under the intact case. The RMSD magnitudes were increased, and they were higher than the CL threshold for the loading cases PS2–PS4, thereby suggesting that the force changes in the anchorage were also alarmed. However, the RMSD indices were less sensitive than the smart rebar–aggregate sensors located inside the concrete anchorage.

#### 4.3.2. Prediction of Compressive Stress via Impedance Signals of Smart Rebar–Aggregate

**Figure 25:**
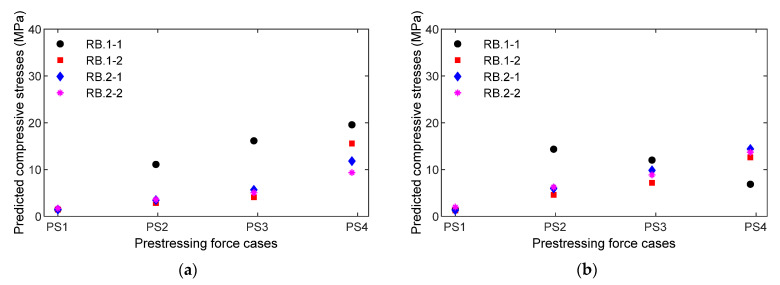
Predicted compressive stresses via measured impedance signatures for loading cases PS1~PS4: four smart rebars RB.1-1~RB.2-2: (**a**) FEM estimation using the empirical equation; (**b**) Experiment prediction.

The empirical equations (see Equations (6)–(9)) of the measured impedance signals with respect to the compressive stresses applied on the concrete cylinder were utilized to predict the compressive stresses in the PSC anchorage subjected to the loading cases PS1~PS4. For the four smart rebars (RB.1-1~RB.2-2) installed in the PSC anchorage, compressive stresses were predicted by applying RMSD values quantified from the measured impedance signatures (see Figure 22) to the empirical equations Equation (6) (for FEM estimation) and Equation (7) (for experimental prediction). Figure 25a shows the prediction of compressive stress via the FEM linear regression equation corresponding to the four loading cases PS1~PS4. Figure 25b shows the prediction of stress via the experimental parabolic regression equation corresponding to the four loading cases PS1~PS4.

**Figure 26:**
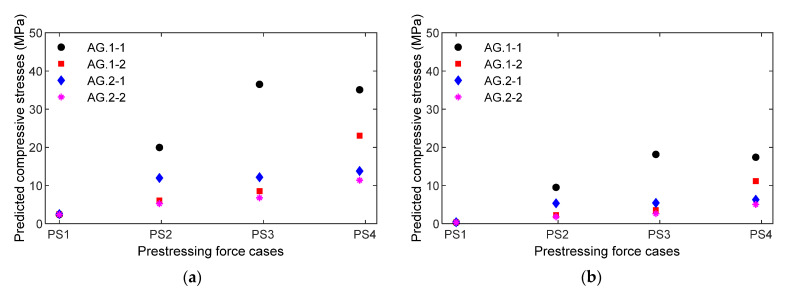
Predicted compressive stresses via measured impedance signatures for loading cases PS1~PS4: four smart aggregates AG.1-1~AG.2-2: (**a**) FEM estimation using the empirical equation; (**b**) Experiment prediction.

For the four smart aggregates (AG.1-1~AG.2-2) installed in the PSC anchorage, the compressive stresses were predicted by applying the measured RMSD values of impedance signals (see Figure 23) to the empirical equations (see Equations (8) and (9). Figure 26a shows the prediction of compressive stress via the FEM linear regression equation (i.e., Equation (8)) corresponding to the four loading cases PS1~PS4. Figure 26b shows the prediction of compressive stress via the experimental parabolic regression equation (i.e., Equation (9)) corresponding to the four loading cases PS1~PS4.

**Figure 27:**
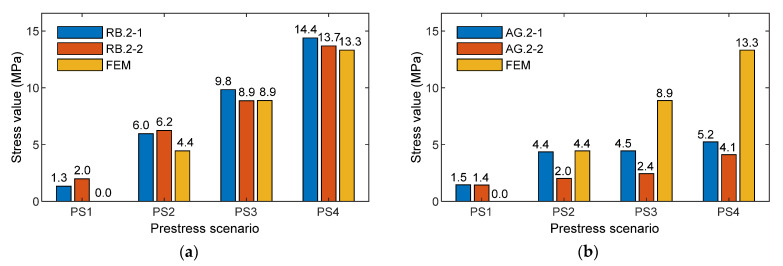
Comparison of stress results between experimental prediction using empirical equation versus numerical simulation of 9-strand anchorage: (**a**) Smart rebar; (**b**) Smart aggregate.

Figure 27 shows a comparison of the stress result between experimental prediction using an empirical equation versus the numerical simulation of the nine-strand anchorage for smart rebars (see Figure 27a) and smart aggregates (see Figure 27b). The PZT sensors mounted on Rebar two were utilized for the evaluation. For the numerical stress simulation, the FE model of the nine-strand anchorage (see Section 2.1) was utilized to obtain stress variations at point L2 (locations of Rebar two in the test) under four loading cases (PS1~PS4). Due to the disturbance of stress distribution in the anchorage, the axial compressive component was selected for the calculation.

These results provide at least four important things. First, the local impedance-based estimation of compressive stress variation was at a single sensor location. For example, the location at smart rebar RB.2-2 varied from 1.98 to 13.7 MPa (see Figure 25b) when the PSC anchorage was subjected to the loading cases PS1~PS4. Second, the impedance-based estimation of local stresses was dependent on the type of smart-sensor materials. For example, the location at smart aggregate AG.2-2 (near smart rebar RB.2-2) varied from 1.44 to 4.11 MPa under the same loading cases. Third, the results give information on relative stress behaviors at different sensor locations in the PSC anchorage. It was noted that the smart rebars and smart aggregates reacted differently to the loading cases, as shown in Figure 25 and Figure 26. Fourthly, numerical stress variation yielded a good agreement of stress estimation using the empirical equation obtained from the smart rebar, thus demonstrating that the practicability of the smart-PZT embedded interface was evaluated.

## 5. Discussion on Smart Rebar–Aggregate Sensor in PSC Anchorage

Prestressing force variation in the nine-strands concrete anchorage was successfully detected using the impedance signatures obtained from the smart rebars, the smart aggregates, and the surface-mounted PZT sensors. The sensitivity of the surface-bonded sensors was less than that of the embedded sensors. It is noted that the bearing plate embedded in concrete led to a significant variation of stresses at its near field as compared to the concrete surface. The result suggests that the smart rebar–aggregate sensors should be implemented for condition monitoring of the anchorage zone.

Comparatively, the PZT-embedded smart rebars gave better indications for prestressing force variations. As shown in Figure 22, the impedance features (i.e., RMSD indices) of the smart rebars were consistently increased as matching the force changes. As shown in Figure 23, the RMSD values of the smart aggregates were not consistent for the magnitudes of prestress forces (e.g., AG.1-1 indicated 5.43% for PS2 but 5.21% for PS 3).

Empirical relationships between the impedance features and the compressive stresses applied were formulated on the basis of the axially loaded concrete cylinder, as shown in Figure 16. The empirical equations were implemented to predict the compressive stresses at the sensor locations in the PSC anchorage (see Figure 25, Figure 26 and Figure 27), subjected to the prestress force scenarios PS1~PS4 with force variation of about 30%. It could be concluded that it is possible to detect and quantify lower stress changes (up to 20%) in the anchorage using smart embedded sensors.

The patterns of impedance features were slightly different for a pair of smart rebar–aggregate sensors (e.g., RB.1-1 and AG.1-1 placed closely in Rebar one), as shown in Figure 22a and Figure 23a. The different patterns would be caused by (1) dissimilar elevations of the smart rebar and the smart aggregate in the concrete anchorage, (2) unequal loadings due to inaccurate manual setups of steel-strands in sequence, and (3) imperfect contact conditions in the bearing plate and concrete body.

Furthermore, the sensitivities of embeddable PZT sensors depend on the material properties of their components (e.g., epoxy, concrete) [22,30]. Thus, an appropriate design of the smart rebar or aggregate should be achieved to detect relatively small stress changes in PSC structures during long-term operation.

## 6. Concluding Remarks

This study investigated the feasibility evaluation of smart PZT-embedded sensors for impedance-based damage monitoring in the PSC anchorage. The concept of impedance-based damage monitoring for the concrete anchorage was concisely introduced. The sample of a PZT-embedded rebar and aggregate was designed for impedance monitoring sensitive to incipient defects in the PSC anchorage. The axially loaded concrete cylinder embedded with the smart rebar–aggregate was numerically and experimentally analyzed to investigate the performance of impedance measurement. Additionally, empirical equations were formulated on the relationships between the measured impedance signatures and the compressive stresses applied. The experimental test on a real-scale anchorage zone was performed to investigate the practicality of smart rebar–aggregate sensors positioned at various locations. For a sequence of loading cases, the variation in impedance responses was quantified to evaluate the accuracy of smart rebar–aggregate sensors. The empirical equations formulated based on the axially loaded concrete cylinder were implemented to predict compressive stresses at sensor locations in the PSC anchorage. 

Based on the results, it can be concluded that (1) the feasibility of the smart rebar–aggregate sensors was successfully evaluated by monitoring the impedance features sensitive to prestress forces; (2) the embedded PZT sensors near the bearing plate yielded higher sensitivity to prestress forces; (3) the empirical equations of the measured impedance signals with respect to the compressive stresses applied could be implemented to predict compressive stresses in the PSC anchorage; and (4) the smart rebar was more sensitive for local monitoring as compared to the smart aggregate.

As a remaining issue, the optimal design parameters of embeddable sensor devices should be achieved to detect relatively small changes of long-term prestress loss induced by concrete creep/shrinkage and steel strands relaxation. Additionally, the effect of temperature variation should be surveyed for the smart rebar–aggregate sensors.

## Figures and Tables

**Table 1:** Concrete mix design for concrete components.

Material for 1 m^3^	Mass (kg)
Water (liter)	165
Sand	800
Cement	346
Aggregate (D_max_ 25)	997

**Table 2:** Material properties of concrete, steel, and epoxy adhesive, PZT 5A.

Properties	Concrete	Steel (Rebar)	Epoxy	PZT 5A
Young’s modulus (GPa)	24.4	200	0.74	62.1
Poisson’s ratio	0.20	0.33	0.38	0.35
Mass density (kg/m^3^)	2400	7850	1090	7750
Compressive strength (MPa)	23.3	-	32.3	-
Yield strength (MPa)	-	390	-	-

## Data Availability

Data available on reasonable request from the corresponding author.
